# Sertoli cell-conditioned medium can improve blood-testis-barrier function and spermatogenesis in azoospermia mice induced by scrotal hyperthermia: An experimental study

**DOI:** 10.18502/ijrm.v22i1.15238

**Published:** 2024-02-23

**Authors:** Fakhroddin Aghajanpour, Reza Soltani, Azar Afshar, Hojjat-Allah Abbaszadeh, Fatemeh Fadaei Fathabadi, Nafiseh Moeinian, Abbas Aliaghaei, Ali Dehghani Nejad, Reza Mastery Farahani, Mohsen Norouzian, Mohammad-Amin Abdollahifar

**Affiliations:** ^1^Department of Biology and Anatomical Sciences, School of Medicine, Shahid Beheshti University of Medical Sciences, Tehran, Iran.; ^2^Student Research Committee, Faculty of Medicine, Shahid Beheshti University of Medical Sciences, Tehran, Iran.; ^3^Department of Biology and Anatomical Sciences, School of Medicine, Qazvin University of Medical Sciences, Qazvin, Iran.

**Keywords:** Testis, Sertoli cells, Culture media, Hyperthermia, Spermatogenesis.

## Abstract

**Background:**

An increase in the temperature of the testis is associated with damage to the epithelium of seminiferous tubules and disruption of sperm production.

**Objective:**

The current study aimed to investigate the effect of the Sertoli cell-conditioned medium (SCCM) on the blood-testis-barrier associated genes and spermatogenesis process following scrotal hyperthermia.

**Materials and Methods:**

In this experimental study, 40 adult NMRI mice (8 wk, 25–30 gr) were allocated into 4 groups: I) control, II) DMEM (10 μl Dulbecco's Modified Eagle Medium), III) scrotal hyperthermia, and IV) scrotal hyperthermia+SCCM (10 μl SCCM). Hyperthermia was induced by placing the mice scrotum in water at 43 C for 20 min every other day for 10 days. Mice were treated every other day for 5 wk. Then the animals were euthanized, and the tails of epididymis were removed to analyze sperm parameters, testis were taken for stereological assessment, reactive oxygen spices and glutathione levels, and the expression of *Ocln*, *Gja1*, *Cdh2*, and *Itgb1*.

**Results:**

The results of sperm analysis indicated that SCCM-treated mice significantly increased sperm count and motility and reduced DNA fragmentation. In addition, histological and molecular findings showed that the volume of testicular tissue, the number of germ cells, the glutathione level, and the expression of *Ocln*, *Gja1*, *Cdh2*, and *Itgb1* genes were significantly increased in the SCCM-treated mice.

**Conclusion:**

Findings suggest that growth factors of SCCM stimulate the proliferation and differentiation of germ cells through paracrine effects and upregulate the blood-testis-barrier-associated genes in mice subjected to scrotal hyperthermia.

## 1. Introduction

The rise in global temperature is currently a public health concern that adversely impacts the body's organs (1). In humans, the activity of the reproductive system is closely dependent on temperature. In most male mammals, the testis, the site of normal spermatogenesis, is located 2–6 C lower than the core body temperature (2). Thus, spermatogenesis is a complex, temperature-dependent, and multistep process that is disturbed by moderate to severe testicular heat stress (3). This stress can be induced by occupational factors like professional driving, medical conditions such as cryptorchidism and obesity, and environmental factors like hot baths and saunas (4).

Within the testicular tissue, a complex interplay between germ and somatic cells takes place (5). Sertoli-supporting cells play a critical role by providing the necessary environment for the differentiation of germ cells into mature spermatozoa. Moreover, Sertoli cells (SCs) form the blood-testis-barrier (BTB), a formidable junctional complex that ensures an immune cell-free environment for germ cell development (6). Maintaining the integrity of this barrier is paramount for spermatogenesis.

Hyperthermia of the scrotum, ranging from mild to severe, has been shown to increase testicular reactive oxygen species (ROS), leading to cell death in both somatic and germ cells. This thermal stress also disrupts the structure of the BTB and the epithelium of seminiferous tubules by altering the expression of proteins in the SCs (7). Research demonstrated that transient hyperthermia could decrease the expression of critical tight junction proteins like claudin-3, zonula occludens-1, and occludin (8). Additionally, heat stress degrades proteins like N-cadherin, disrupting communication between adjacent SCs and the integrity of the spermatogenic epithelium (9). Consequently, damage to the SCs leads to BTB disintegration and a reduction in sperm production.

SCs are pivotal in sperm production and fertility, as they secrete paracrine factors, cytokines, and nutrients. They also release various inductive proteins, including insulin-like growth factor I (IGF-I), transforming growth factor α, fibroblast growth factor (FGF), glial cell-derived neurotrophic factor, and stem cell factor, which stimulate spermatogonial stem cells' proliferation and germ cell differentiation (10, 11). In vitro studies have demonstrated that SCs-conditioned medium (SCCM) could induce mesenchymal stem cells into the germ line (12). Additionally, SCCM has shown promise in improving spermatogenesis in animal models of testicular injury (13).

This experiment's hypothesis centers around the potential of SCCM to improve BTB function and spermatogenesis through the secretion of growth factors and regulators. Thus, this study was designed to evaluate the effects of SCCM on spermatogenesis and the retrieval of BTB-related genes following scrotal hyperthermia in a mice model.

## 2. Materials and Methods

### Animals

In the present experimental study, 40 adult NMRI mice (8 wk, 25–30 gr) were purchased from the experimental animal center, Royan Institute, Tehran, Iran, and were kept in a clean cage with control condition according to the National Institutes of Health guideline (12 hr light/dark cycle, room temperature: 22 ± 3 C). All animals had free access to water and food.

Dulbecco's Modified Eagle Medium F12 (DMEM), fetal bovine serum, and lysis buffer were purchased from ABCAM (Cambridge, UK). DCFDA, trypsin, collagenase, and Hams F-10 were obtained from sigma Aldrich (St. Louis, MO, USA). Polymerase chain reaction kit was purchased from Takara (TAKARA, Bio, Japan). SCD kit was obtained from Indas labratorios (INDAS Lab, Madrid, Spain). DNaseI was purchased from Roche (Roche, Basel, Switzerland). Glutathione assay kit was obtained from Zellbio (Zellbio, Lonsee, Germany). Other materials were purchased from Santa Cruz Biotechnology (Santa Cruz, CA, USA).

### Induction of scrotal hyperthermia

The animals were anesthetized with intraperitoneal injections of ketamine (100 mg/kg) and xylazine (5 mg/kg). For 10 days, the mice's scrotums were exposed to a temperature of 43 C for 20 min every other day. Following the induction of scrotal hyperthermia, the animals were dried and caged until they regained consciousness (14). After induction of scrotal hyperthermia, some rats required more time to recover.

### Study design 

The sample size was selected based on the previous study (15). The study utilized randomization for allocating experimental units to control and treatment groups. The randomization was performed based on the weight of the animals, where all animals were weighed, and the randomization sequence was generated according to their weights. This method was employed to ensure a balanced distribution of weights across the groups. All mice were allocated to 4 groups (n = 10/each): I) control (Cont), II) DMEM, III) scrotal hyperthermia (Hyp), and IV) scrotal hyperthermia + SCCM. The animals in the Cont groups were intact. In groups II, III, and IV, the scrotum of the animals was placed in water at 43 C for 20 min every other day for 10 days. Subsequently, animals in groups II and IV received DMEM (10 μl) and SCCM (10 μl), respectively, intraperitoneally every other day for 5 wk. The exclusion criteria of this study included scrotal inflammation and genital abnormalities. Only the corresponding authors and the animal model operator were aware of the groups, and for other experiments performed, experts were blinded. The outcome criteria evaluated in this research included sperm parameters (sperm count, motility, and DNA fragmentation), testicular histology (volume of testicular tissue and the number of germ cells), biochemical markers (ROS and glutathione [GSH] levels), and molecular markers (expression levels of *Ocln*, *Gja1*, *Cdh2*, and *Itgb1* genes at the mRNA level).

The primary outcome measure for hypothesis testing and sample size determination in this study is the presence or absence of azoospermia. Azoospermia, defined as the absence of sperm in the ejaculate, is a critical variable central to the study investigation into the effects of scrotal hyperthermia and SCCM on spermatogenesis. This measure is pivotal for assessing the impact on sperm parameters and serves as the main focus for testing the study hypothesis.

### Isolation and culture of SCs 

**Figure 1:**
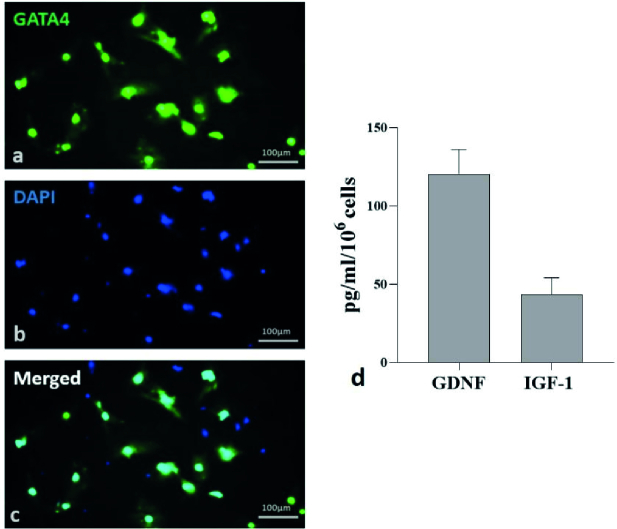
Characterization of mice Sertoli cells (SCs). a-c) Photomicrograph indicated that the immunofluorescence of GATA4 (green) is expressed in SCs and counterstained with DAPI (blue). d) Serum-free SCCM was analyzed for some specific growth factors by ELISA. Data were presented as Mean ± SD (n = 4). GDNF: Glial cell line-derived neurotrophic factor, IGF-1: Insulin-like growth factor-1.

In order to prepare a conditioned medium, SCs were isolated from the testis of 28-day-old male mice. By cervical dislocation, mice were euthanized, and their testes were removed. After decapsulation, 0.25% trypsin (Sigma) and 0.1% collagenase (Sigma, type IV) were used for 15 min at 37 C to finish the enzymatic digestion process. In previous studies, cells were isolated, transferred to T75 cell culture flasks, and kept in an incubator at 37 C, 85 H, and 5 C (CO ${\;}_{2}$ : 5%) (13). The culture medium was changed, and the cells were washed after 24 hr. To obtain SCs with a purity > 96%, the cells were treated with 20 mM Tris-HCL (pH 7.4) for 2.5 min after 48 hr. After washing 3 times with phosphate-buffered saline, serum-free DMEM media were added. Cells were incubated for 48 hr, and the conditioned media was stored at -80. The SCs were marked by GATA4 immunofluorescence (Figure 1, Table I).

### Sperm parameters

In order to analyze the parameters of the sperm, the epididymal tail was removed, minced, and transferred to a petri dish containing 1 ml of Ham's F-10 medium (Sigma-Aldrich Product No. N6635). During an incubation period of 15–20 min, the dishes were placed in an incubator at 37 C. Then, 10 µl of each sample was placed on a slide, and sperm motility was observed with an optical microscope. A hemocytometer slide was used to count the sperm. The number of sperm was counted using a hemocytometer slide.

### Sperm chromatin dispersion (SCD) test

The SCD test was conducted according to the instructions required by the Halospermt kit (INDAS Laboratories, Madrid, Spain). A halo is not visible around the head of sperm with fragmented DNA, but a halo can be seen in healthy sperm. A minimum of 300 sperm were scored in each sample under a × 100 light microscope (14).

### Tissue preparation

24 hr after the end of the injection period, mice of all groups were anesthetized with ketamine and xylosin, and their testis were removed. The right testis was placed in Bouin's solution for 48 hr. After fixation, the specimens were transferred to a tissue processor and then embedded in paraffin. From each sample, serial sections with 5 and 20 μm thickness were prepared for stereological techniques and stained with hematoxylin and eosin (H&E).

### Estimating of testicular volume

The total volume of the testis was assessed by the Cavalieri method and the following formula (14): 


$$V=\sum P\times t\times \frac{a}{p}$$

In this equation, the ΣP is the number of points counted, and the a/p is the area of probe points divided by the magnification. The t is the distance between tissue sections.

### Estimating of the number of testicular cells

Based on the optical dissector method and the following formula, the number of testicular cells was estimated (14): 


$$N_{V}=\frac{\sum_{i=1}^{n}Q}{h\times \sum P\times a/f}\times \frac{t}{B.A}$$

In the formula mentioned above, ΣQ, which represents the count of testicular cells, was determined with a counting frame probe and a microcator (Heidenhain, Germany) connected to the microscope stage. The microcator was used to measure both the height of the dissector (h) and the real thickness of the tissue section (t). Σp denotes the overall count of fields that were included in the counting process. The term a/f stands for the probe area divided by the magnification factor, while BA represents the thickness of the tissue section.

### ROS and GSH production

The ROS content of testicular tissue was measured using dichlorofluorescin diacetate (DCFDA: CAS Number 2044–85-1, Sigma-Aldrich, USA) and spectrofluorimetric method. In this test, 50 mg of tissue was mixed with 100 μL of 20 μM DCFDA. The samples were then incubated for 45 min at 37 C in the dark. After incubation, the testicular tissue was cut into small pieces with a sonicator. Next, the lysed samples were centrifuged at 1500 rpm for 5 min at 4 C. Then, the supernatant of the samples was checked using a spectrofluorimeter with a wavelength of 488 nm. To analyze the data with logarithmic concentrations, 5 concentrations were calculated and a standard graph was drawn. Other samples were evaluated using a standard chart. The ROS level was determined as mM H ${\;}_{2}$ O ${\;}_{2}$ based on the standard curve. According to the manufacturer's instructions, Glutathione peroxidases was determined using a glutathione assay kit (Zellbio, Lonsee, Germany). 1 molar of GSH is decomposed by 1 Glutathione peroxidases activity unit in 1 min. At 495 nm excitation and 530 nm emission wavelengths, 2 markers named O-phthalaldehyde and N-Ethylmaleimide were applied in the test to determine the ratio (16).

### Analysis of *Ocln*, *Gja1*, *Cdh2*, and *Itgb1* expression using real-time polymerase chain reaction

After removing the left testis, the testis tissues were immediately transferred and stored at -80 C. After RNA samples were extracted, they were treated with DNaseI (Roche, Basel, Switzerland) to eliminate genomic DNA contamination. The cDNA was synthesized at 42 C for 60 min using Fermentas' commercial kit. The relative gene expression was quantified by real-time PCR (TaqMan) using QuantiTect SYBR Green Real-Time Polymerase Chain Reaction kit (Takara Bio Inc., Japan). The prepared microtubes containing the PCR reaction were placed in the machine after mixing. Data including CT numbers, threshold cycle, and melting and proliferation curves for each gene were analyzed. The CT numbers related to the reference gene and the main genes of each sample were calculated in the formula 2 ${\;}^{-\Delta \Delta Ct}$ , relative expression changes of each gene.

Primer 3 Plus was used to design forward and reverse primer pairs. The primer-blast tool was used to test PCR primers designed with Primer 3 Plus software prior to the experiment. The housekeeping gene glyceraldehyde 3-phosphate dehydrogenase was considered an internal control (Table II: Primer design).

**Table 1:** Descriptive statistics of the ELISA test


**Factors**	**Mean ± SD (Std. error of mean)**	**Median**	**IQR**
**GDNF**	120.6 ** ± ** 15.34 (8.857)	115.9	29.6
**IGF-1**	43.45 ** ± ** 10.75 (6.205)	44.20	21.46
All data of this table analyzed by *t* test. GDNF: Glial cell line-derived neurotrophic factor, IGF-1: Insulin-like growth factor-1			

**Table 2:** Primer design


**Genes**	**Primer sequences**	**Product length (bp)**
	Forward: AGTTGTGGGAGAAGGGAGAGG	
*Ocln*	Reverse: ACTGGAGATAGGAAAGTGATGGA	156
			Forward: TGCTATGACAAGTCCTTCCCC	
*Gja1*	Reverse: TGCCGTGTTCTTCAATCCCAT	238
			Forward: CCACCTCCGTATCCTCCATCC	
*Cdh2*	Reverse: GCGCGAGTGTGTGTGTATGTG	551
			Forward: CACCCGAGACCAACCGAGAAG	
*Itgb1*	Reverse: CCAATCCAGGAAACCAGTTGC	181
			Forward: CAGAACATCATCCCAGCCTCC	
*GAPDH*	Reverse: TTGGCAGGTTTCTCAAGACGG	152

### Ethical considerations

The procedures were approved by the Research Ethics Committees Laboratory Animals, Shahid Beheshti University of Medical Sciences, Tehran, Iran (Code: IR.SBMU.ACE.1401.013). In the present experimental research, the ethical protocols of the guidelines for working with laboratory animals were observed.

### Statistical analysis

Shapiro-Wilk test was used to check the normality of the data. The data were analyzed by one-way ANOVA test and Duncan Post Hoc using SPSS version 21 software (SPSS, Chicago, IL, USA). The results are presented as mean ± standard deviation. A significance level of p < 0.05 was used. The Kruskal-Wallis test was used if the data were not normal.

## 3. Results

### Sperm parameters

**Figure 2:**
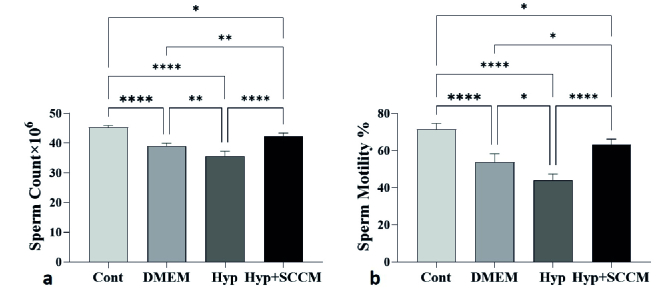
Sperm parameters increased in scrotal hyperthermia-induced mice after injection of SCCM. a) sperm count and b) sperm motility. *P < 0.05, **P < 0.01, and ****P < 0.0001. All values were expressed as Mean ± SD.

The results of semen analysis showed that the sperm count and motility was significantly decreased in the Hyp group compared to the Cont group (p < 0.0001 and p < 0.0001, respectively). In SCCM-treated mice, these parameters significantly increased compared to the Hyp group (p < 0.0001 and p < 0.0001, respectively) and the DMEM group (p < 0.01 and p = 0.01, respectively) (Figure 2, Table III).

### SCD index

**Figure 3:**
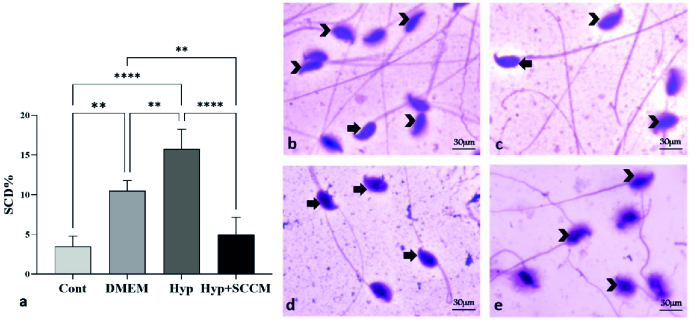
a) Injection of SCCM reduced SCD-positive cells in scrotal hyperthermia-induced mice. **P < 0.01 and ****P < 0.0001. All data were presented as Mean ± SD (n = 6). Photomicrograph of the sperm chromatin dispersion (SCD) with SCD kit staining, × 100. Sperm DNA fragmentation (arrows), healthy sperm (arrowhead). b) Cont, c) DMEM, d) Hyp, and e) Hyp+SCCM.

The results showed that the percentage of SCD-positive sperm was significantly increased in the Hyp group compared to the Cont group (p < 0.0001). A significant decrease in the number of SCD-positive cells was observed in the Hyp+SCCM group compared to the Hyp group (p < 0.0001) and the DMEM group (p < 0.01). No significant difference was observed between the Cont group and the Hyp+SCCM group (Figure 3, Table III).

### Total volume of the testis

**Figure 4:**
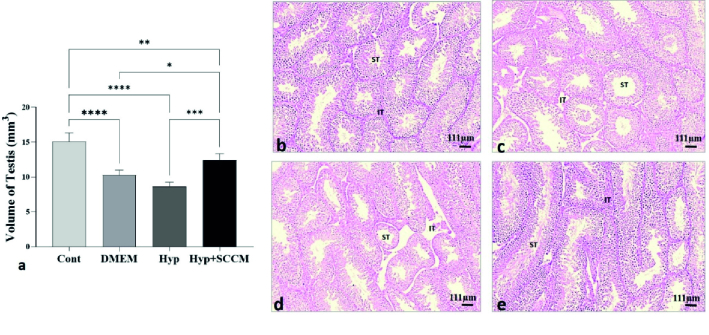
a) Volume of testicular tissue was increased in SCCM-treated mice. *P < 0.05, **P < 0.01, ***P < 0.001, and ****P < 0.0001. All values were expressed as Mean ± SD (n = 5). Photomicrograph of the testicular tissue stained with H&E × 10. b) Cont, c) DMEM, d) Hyp, and e) Hyp+SCCM. ST: Seminiferous tubules, IT: Interstitial tissue.

Based on the data analysis, testicular tissue volume was reduced in the mice in the Hyp group compared to the Cont group (p < 0.0001). The testis volume improved significantly after SCCM injection compared to the Hyp group (p < 0.001) and the DMEM group (p = 0.02) (Figure 4, Table III).

### Number of testicular cells

**Figure 5:**
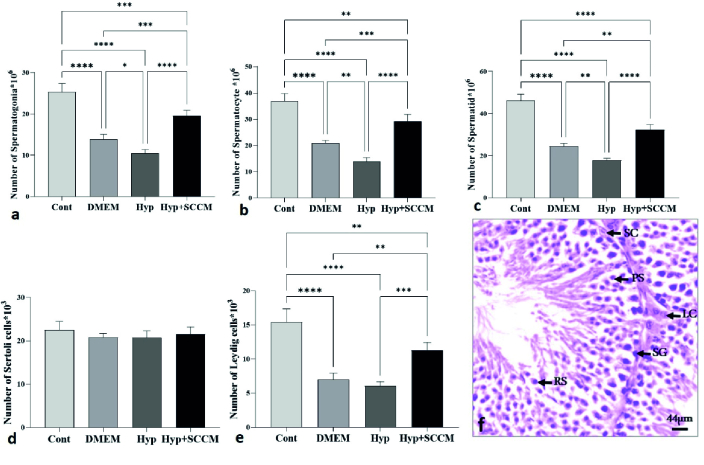
The total number of germ cells in the testis tissue was increased in SCCM-treated mice. a) spermatogonia, b) spermatocyte, c) spermatid, d) Leydig, and e) Sertoli. *P < 0.05, **P < 0.01, ***P < 0.001, and ****P < 0.0001. All data were expressed as Mean ± SD (n = 5). f) Photomicrograph of the testis stained with H&E, × 40. SG: Spermatogonia, PS: Primary spermatocyte, RS: Round spermatid, SC: Sertoli cell, LC: Leydig cell.

The results of stereological assessments in the present study revealed a significant decrease in the number of spermatogonia, spermatocytes, spermatids, and Leydig cells in the Hyp group compared to the Cont group (p < 0.0001, p < 0.0001, p < 0.0001, and p < 0.0001, respectively). The results indicated that the number of these cells in the Hyp+SCCM group was significantly higher than the Hyp group (p < 0.0001, p < 0.0001, p < 0.0001, and p < 0.001, respectively) and the DMEM group (p < 0.001, p < 0.001, p < 0.01, and p < 0.01). No significant difference was observed in the number of SCs between the study groups (Figure 5, Table III).

### Total ROS and GSH content of the testis

**Figure 6:**
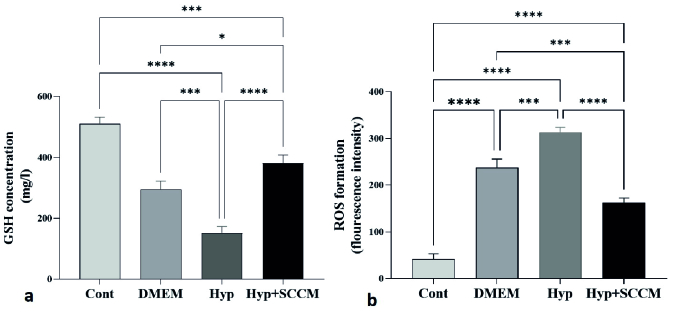
In the testis of SCCM-treated mice, reactive oxygen spices (ROS) formation decreased, and glutathione (GSH) content increased. a) GSH content and b) ROS formation. *P < 0.05, ***P < 0.001, and ****P < 0.0001. All data were expressed as Mean ± SD (n = 4).

The results showed a significant formation of the ROS in the Hyp group compared to the Cont group (p < 0.0001). However, ROS production was significantly decreased in the testis tissue of the Hyp+SCCM group compared to the Hyp group (p < 0.0001) and DMEM group (p < 0.001) (Figure 6, Table III). Based on the findings, the GSH content in the Hyp group was remarkably lower compared to the Cont group (p < 0.0001). GSH production in the Hyp+SCCM group significantly increased compared to the Hyp group (p < 0.0001) and DMEM group (p = 0.01) but significantly decreased compared to the Cont group (p < 0.001) (Figure 6, Table III).

### Real-time PCR analysis

**Figure 7:**
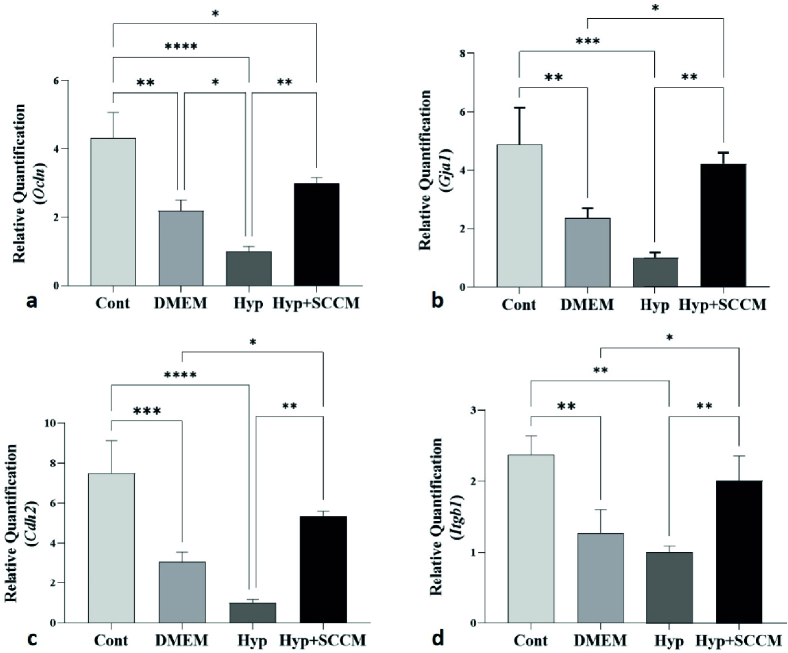
Injection of SCCM upregulated the expression of BTB-associated genes in scrotal hyperthermia-induced mice. a) Ocln, b) Gja1, c) Cdh2, and d) Itgb1. *P < 0.05, **P < 0.01, ***P < 0.001, and ****P < 0.0001. All values were expressed as Mean ± SD (n = 5).

The relative mRNA expression of *Ocln*, *Gja1*, *Cdh2*, and *Itgb1* in testicular tissue was examined, and the results showed that the remarkably downregulated in scrotal hyperthermia-induced mice compared to the Cont group (p < 0.0001, p < 0.001, p < 0.001, and p < 0.01, respectively). SCCM injection significantly upregulated the gene expression compared to the Hyp group (p < 0.01, p < 0.01, p < 0.01, and p < 0.01, respectively). The relative mRNA expression levels of the tested genes (*Ocln*, *Gja1*, *Cdh2*, and *Itgb1*) among the groups are depicted in figure 7, and table IV.

**Table 3:** Descriptive statistics


	**Groups**				
**Variable**	**Cont**	**DMEM**	**Hyp**	**Hyp+SCCM**	**P-value**
	**Sperm count**	45.25 ± 0.6758 (45.30, 1.3)	39.00 ± 0.9345 (39.05, 1.8)	35.65 ± 1.636 (35.40, 3.05)		42.25 ± 1.133 (42.05, 2.1)	< 0.0001 ${\;}^{\text{a}}$ < 0.0001 ${\;}^{\text{b}}$ 0.01 ${\;}^{\text{c}}$ < 0.01 ${\;}^{\text{d}}$ < 0.01 ${\;}^{\text{e}}$ < 0.0001 ${\;}^{\text{f}}$
**Sperm motility**	71.50 ± 3.109 (71.50, 6)	53.75 ± 4.573 (54.00, 8.75)	44.00 ± 3.367 (44.00, 7.5)	63.00 ± 3.162 (63.50, 6)	< 0.0001 ${\;}^{\text{a}}$ < 0.0001 ${\;}^{\text{b}}$ 0.02 ${\;}^{\text{c}}$ 0.01 ${\;}^{\text{d}}$ 0.01 ${\;}^{\text{e}}$ < 0.0001 ${\;}^{\text{f}}$
**SCD**	3.500 ± 1.291 (3.500, 2.5)	10.50 ± 1.291 (10.50, 2.5)	15.75 ± 2.500 (15.50, 4.75)	5.00 ± 2.160 (4.500, 4)	0.0010 ${\;}^{\text{a}}$ < 0.0001 ${\;}^{\text{b}}$ 0.68 ${\;}^{\text{c}}$ < 0.01 ${\;}^{\text{d}}$ < 0.01 ${\;}^{\text{e}}$ < 0.0001 ${\;}^{\text{f}}$
**Testis volume**	15.10 ± 1.219 (14.80, 2.25)	10.28 ± 0.713 (10.30, 1.375)	8.65 ± 0.624 (8.700, 1.2)	12.45 ± 0.896 (12.25, 1.65)	< 0.0001 ${\;}^{\text{a}}$ < 0.0001 ${\;}^{\text{b}}$ < 0.01 ${\;}^{\text{c}}$ 0.09 ${\;}^{\text{d}}$ 0.02 ${\;}^{\text{e}}$ 0.0003 ${\;}^{\text{f}}$
**Number of spermatogonia**	25.37 ± 2.017 (24.72, 3.61)	13.86 ± 1.239 (13.85, 5.21)	10.54 ± 0.829 (10.81, 1.42)	19.59 ± 1.350 (19.95, 2.48)	< 0.0001 ${\;}^{\text{a}}$ < 0.0001 ${\;}^{\text{b}}$ < 0.001 ${\;}^{\text{c}}$ 0.02 ${\;}^{\text{d}}$ < 0.001 ${\;}^{\text{e}}$ < 0.0001 ${\;}^{\text{f}}$
**Number of primary spermatocytes**	36.94 ± 2.832 (37.42, 5.3)	20.97 ± 0.950 (21.23, 1.75)	13.92 ± 1.421 (13.68, 1.7)	29.29 ± 2.510 (29.06, 4.84)	< 0.0001 ${\;}^{\text{a}}$ < 0.0001 ${\;}^{\text{b}}$ 0.001 ${\;}^{\text{c}}$ < 0.01 ${\;}^{\text{d}}$ < 0.001 ${\;}^{\text{e}}$ < 0.0001 ${\;}^{\text{f}}$
**Number of spermatids**	46.18 ± 3.077 (46.50, 5.76)	24.49 ± 1.444 (24.71, 2.73)	17.92 ± 0.821 (17.94, 1.53)	32.32 ± 2.555 (31.34, 4.37)	< 0.0001 ${\;}^{\text{a}}$ < 0.0001 ${\;}^{\text{b}}$ < 0.0001 ${\;}^{\text{c}}$ < 0.01 ${\;}^{\text{d}}$ < 0.01 ${\;}^{\text{e}}$ < 0.0001 ${\;}^{\text{f}}$
**Number of Leydig**	15.38 ± 1.957 (15.25, 3.75)	6.986 ± 0.947 (7.044, 1.83)	6.074 ± 0.613 (6.044, 1.19)	11.27 ± 1.179 (11.36, 2.27)	< 0.0001 ${\;}^{\text{a}}$ < 0.0001 ${\;}^{\text{b}}$ < 0.01 ${\;}^{\text{c}}$ 0.74 ${\;}^{\text{d}}$ < 0.01 ${\;}^{\text{e}}$ < 0.001 ${\;}^{\text{f}}$
**Number of Sertoli**	22.51 ± 1.952 (22.12, 3.65)	20.79 ± 0.903 (20.87, 1.73)	20.71 ± 1.587 (20.58, 3)	21.55 ± 1.644 (21.64, 3.14)	0.44 ${\;}^{\text{a}}$ 0.4 ${\;}^{\text{b}}$ 0.82 ${\;}^{\text{c}}$ 0.99 ${\;}^{\text{d}}$ 0.9 ${\;}^{\text{e}}$ 0.87 ${\;}^{\text{f}}$
**ROS**	41.98 ± 11.01 (43.55, 21.85)	237.6 ± 18.28 (239.6, 36.4)	313.7 ± 9.993 (317.0, 19.1)	162.2 ± 10.26 (163.9, 20.3)	< 0.0001 ${\;}^{\text{a}}$ < 0.0001 ${\;}^{\text{b}}$ < 0.0001 ${\;}^{\text{c}}$ < 0.001 ${\;}^{\text{d}}$ < 0.001 ${\;}^{\text{e}}$ < 0.0001 ${\;}^{\text{f}}$
**GSH**	511.3 ± 21.38 (513.6, 42.5)	295.6 ± 26.99 (297.8, 53.9)	152.4 ± 20.22 (154.0, 40.4)	381.3 ± 26.48 (387.2, 52.7)	< 0.0001 ${\;}^{\text{a}}$ < 0.0001 ${\;}^{\text{b}}$ < 0.001 ${\;}^{\text{c}}$ < 0.001 ${\;}^{\text{d}}$ 0.01 ${\;}^{\text{e}}$ < 0.0001 ${\;}^{\text{f}}$
Data presented as Mean ± SD (median, IQR). One-Way ANOVA and multiple Duncan. a: Cont vs DMEM, b: Cont vs Hyp, c: Cont vs Hyp+SCCM, d: DMEM vs Hyp, e: DMEM vs Hyp+SCCM, f: Hyp vs Hyp+SCCM, Cont: Control, DMEM: Dulbecco's Modified Eagle Medium, Hyp: Scrotal hyperthermia, SCCM: Sertoli cell-derived conditioned medium, SCD: Sperm chromatin dispersion, ROS: Reactive oxygen spices, GSH: Glutathione					

**Table 4:** Descriptive statistics of genes


	**Groups**				
**Genes**	**Cont**	**DMEM**	**Hyp**	**Hyp+SCCM**	**P-value**
	*Ocln*	4.317 ± 0.750 (4.144, 1.47)	2.193 ± 0.317 (2.135, 0.627)	1.007 ± 0.139 (1.080, 0.248)		2.992 ± 0.166 (3.023, 0.328)	< 0.01 ${\;}^{\text{a}}$ < 0.0001 ${\;}^{\text{b}}$ 0.02 ${\;}^{\text{c}}$ 0.03 ${\;}^{\text{d}}$ 0.17 ${\;}^{\text{e}}$ < 0.01 ${\;}^{\text{f}}$
*Gja1*	4.885 ± 1.259 (4.951, 2.515)	2.358 ± 0.347 (2.350, 0.693)	1.010 ± 0.175 (0.934, 0.326)	4.229 ± 0.374 (4.436, 0.657)	< 0.01 ${\;}^{\text{a}}$ < 0.001 ${\;}^{\text{b}}$ 0.65 ${\;}^{\text{c}}$ 0.15 ${\;}^{\text{d}}$ 0.04 ${\;}^{\text{e}}$ < 0.01 ${\;}^{\text{f}}$
*Cdh2*	7.514 ± 1.614 (7.764, 3.199)	3.059 ± 0.489 (3.128, 0.971)	1.008 ± 0.157 (0.984, 0.313)	5.355 ± 0.248 (5.285, 0.482)	< 0.01 ${\;}^{\text{a}}$ < 0.0001 ${\;}^{\text{b}}$ 0.05 ${\;}^{\text{c}}$ 0.07 ${\;}^{\text{d}}$ 0.04 ${\;}^{\text{e}}$ < 0.01 ${\;}^{\text{f}}$
*Itgb1*	2.375 ± 0.261 (2.290, 0.502)	1.274 ± 0.326 (1.341, 0.644)	1.002 ± 0.084 (1.047, 0.105)	2.011 ± 0.345 (2.103, 0.672)	< 0.01 ${\;}^{\text{a}}$ < 0.01 ${\;}^{\text{b}}$ 0.41 ${\;}^{\text{c}}$ 0.63 ${\;}^{\text{d}}$ 0.04 ${\;}^{\text{e}}$ < 0.01 ${\;}^{\text{f}}$
Data presented as Mean ± SD (median, IQR). One-Way ANOVA. a: Cont vs DMEM, b: Cont vs Hyp, c: Cont vs Hyp+SCCM, d: DMEM vs Hyp, e: DMEM vs Hyp+SCCM, f: Hyp vs Hyp+SCCM, Cont: Control, DMEM: Dulbecco's Modified Eagle Medium, Hyp: Scrotal hyperthermia, SCCM: Sertoli cell-derived conditioned medium					

## 4. Discussion

This study aimed to investigate the effect of SCCM on sperm parameters, testicular tissue, and *BTB*-associated gene expression after scrotal hyperthermia. The results showed that scrotal hyperthermia decreases the sperm count and motility, testicular volume, GSH level, and expression of *Ocln*, *Gja1*, *Cdh2*, and* Itgb1* genes and increases the production of ROS and sperm DNA fragmentation. Consistent with previous findings, scrotal hyperthermia mediates cell death by producing reactive substances and depleting antioxidants, resulting in atrophy of testicular tissue by reducing the number of germ cells (14). In the seminiferous tubules, SCs ensure the survival of spermatogenic cells by producing and secreting a variety of factors and forming a nutritional and supportive network. In addition, SCs protect testicular cells against oxidative stress by producing GSH (17).

The stereological results of this study showed that the number of spermatogonia, spermatocytes, and spermatids cells increased after the injection of the SCCM. This finding may be the presence of some paracrine factors, such as glial cell-derived neurotrophic factor and stem cell factor, which can regulate the growth of testicular tissue after injury by stimulating the development of germ cells (18, 19). A significant increase in the number of Leydig cells was also observed. SCs appear to stimulate Leydig cell proliferation by secreting transforming growth factor α (17). This study found no significant differences in the number of SCs between the groups, possibly due to the heat stress resistance of these cells (14). However, IGF-I in the SCCM enhances SC function and protein expression with an autocrine effect via the IGF-I receptor (20). Another finding of this experiment was an increase in sperm DNA fragmentation after scrotal hyperthermia. Overformation of reactive substances, poor antioxidant capability, and defective maturation are sources of damage to sperm DNA, which leads to a decrease in semen quantity and quality (21). It was found that the above parameter was improved in SCCM-treated mice. SCc protects sperm through various paracrine factors. Among these factors, IGF-I can stabilize DNA by regulating spermatozoa maturation, which then increases sperm motility and count (22). These findings are consistent with previous studies. Panahi et al. reported that sperm parameters, testicular volume, and cell numbers were improved after the injection of SCCM in a busulfan-induced azoospermia mice model (13). In this way, SCs reduce testicular damage by secreting growth factors.

The results of real-time PCR tests indicated that scrotal hyperthermia downregulated the expression of *Ocln*, *Gja1*,* Cdh2*, and *Itgb1*. The maintenance of BTB structure depends on the expression of genes and proteins of tight, desmosomes, ectoplasmic specialization, and gap junctions (23). *Ocln* is an occludin coding gene, one of the most important tight junction proteins. Reducing its expression increases the permeability of the BTB and the access of immune cells to the differentiating spermatocytes, which leads to a decrease in the number of germ cells and a decrease in the sperm parameters (24). *Cdh2* is an N-cadherin coding gene and expressed by germ cells and SCs and plays an important role in intercellular interaction, the integrity of the spermatogenic epithelium, and the maintenance of tight junctions (25). *Gja1* establishes a cross talk between adjacent SCs by expressing connexin43, the widest gap junction protein, and regulates barrier homeostasis. *Itgb1* (* β 1* integrin coding gene) localized to the apical ectoplasmic specialization, plays a role in the maturation of elongated spermatids (24). Recent studies suggest that oxidative stress is the initiating factor for BTB disintegration.

In vitro evidence revealed overproduction of ROS suppress expression of *Gja1* and *Itgb1* in SCs can lead to germ cells losses (26). In addition, previous research demonstrated that heat stress changes the BTB dynamics. A study by Yang et al., showed that ROS formation in SCs following heat shock downregulated the expression of Claudin-11, JAM-A, Occludin, and zonula occludens-1 at the mRNA and protein level (27). Consistent with the previous evidence, the results of this study showed that scrotal hyperthermia reduces the *BTB*-associated gene expression by stimulating oxidative stress. According to the results, expression of *Ocln*, *Gja1*, *Cdh2*, and *Itgb1* genes were improved after injection of SCCM. Factors that regulate SCs secretion appear to act autocrine on these cells and regulate their activity at the molecular level. For instance, basic FGF is expressed and secreted by SCs. Basic FGF maintains the survival of SCs and interactions between these cells with spermatogenic and peritubular cells. In addition, it regulates SCs function through an autocrine effect (28).

Furthermore, the SCs secrete nerve growth factor. The nerve growth factor receptor is expressed on the surface of SCs and increases their survival. This factor also restores the expression of the zonula occludens-1, *Ocln*, *Gja1*, and *Cdh2* genes and the dynamics of BTB after testicular injury (29). The upregulation of gene expression in the present study could be due to the presence of these factors in SCCM. Based on the results of this study, the function of the testicular tissue and the spermatogenesis could be improved after injection of SSCM in scrotal hyperthermia mice model. In this research, sperm parameters, genes related to BTB, and testicular tissue were investigated. However, some limitations should be considered. Including the expression of proteins related to BTB was not evaluated.

## 5. Conclusion

The results of the present study clearly showed that SCCM by stimulating the spermatogonial stem cells self-renewal, spermatocyte meiosis, and the expression of *Ocln*, *Gja1*, *Cdh2*, and *Itgb1* genes through paracrine and autocrine factors plays a crucial role in regulating the BTB and protects the spermatogenesis process.

##  Data availability

The data and materials supporting this study's findings are available from the corresponding author upon reasonable request.

##  Author contributions

Mohsen Norouzian and Mohammad-Amin Abdollahifar had full access to all of the data in the study and takes responsibility for the integrity of the data and the accuracy of the data analysis.

Concept and design Mohsen Norouzian, Mohammad-Amin Abdollahifar, and Hojjat-Allah Abbaszadeh.

Acquisition, analysis, or interpretation of data Fakhroddin Aghajanpour, Reza Soltani, Azar Afshar, Fatemeh Fadaei Fathabadi, Nafiseh Moeinian, Abbas Aliaghaei, Ali Dehghani Nejad, and Reza Mastery Farahani.

Drafting of the manuscript: Fakhroddin Aghajanpour, Reza Soltani, Azar Afshar, Hojjat-Allah Abbaszadeh, Fatemeh Fadaei Fathabadi, Nafiseh Moeinian, Abbas Aliaghaei, Ali Dehghani Nejad, and Reza Mastery Farahani.

Critical revision of the manuscript for important intellectual content: All authors.

Statistical analysis: Fakhroddin Aghajanpour, Reza Soltani, Azar Afshar, Hojjat-Allah Abbaszadeh, Fatemeh Fadaei Fathabadi, Nafiseh Moeinian, Abbas Aliaghaei, Ali Dehghani Nejad, and Reza Mastery Farahani.

Supervision: Mohsen Norouzian and Mohammad-Amin Abdollahifar.

##  Conflict of Interest 

The authors declare that there is no conflict of interest.
